# Supplementary material for: Reflected generalized concentration addition and Bayesian hierarchical models to improve chemical mixture prediction
Source: PLoS One. 2024 Mar 28;19(3):e0298687. doi: 10.1371/journal.pone.0298687 (PMC10977799; doi:10.1371/journal.pone.0298687)
Supplement: S1 File — (PDF) [file pone.0298687.s001.pdf]

Supplementary Information: Reflected Generalized Concentration Addition and Bayesian Hierarchical Models to Improve Chemical Mixture Prediction

Daniel Zilber[1] and Kyle P Messier[1]

[1]National Institute of Environmental Health Sciences, Division of Translational Toxicology

March 7, 2024

1 RGCA Details

1.1 RGCA formula for Negative sill

The piecewise inverse RGCA function for the case of a negative sill is provided here:

$$c = f^{-1}(r|\alpha < 0, \theta, \beta > 0) = \begin{cases} -2\theta + \frac{\theta}{1+\left(\frac{\alpha}{r-2\alpha}\right)^{1/\beta}} & r \in (-\infty, 2\alpha) \\ -2\theta - \theta\left(\frac{\alpha}{2\alpha - r} - 1\right)^{-1/\beta} & r \in (2\alpha, \alpha) \\ \theta\left(\frac{\alpha}{r} - 1\right)^{-1/\beta} & R \in [\alpha, 0) \\ -\frac{\theta}{1+\left(\frac{-\alpha}{r}\right)^{1/\beta}} & r \in (0, \infty) \end{cases} \tag{1}$$

1.2 Isobolograms

We illustrate the effect of different relative values of a sill α and slope β in a simulated binary mixture in figure 1. The top row shows different sill values while the right column shows different slope values. The reference chemical has a sill $\alpha = 5$, $EC_{50}$of $\theta = 1$ and slope $\beta = 1$,

while the $EC_{50}$for our alternate chemical is $\theta = 0.7$. When the sills and slopes are equal we recover CA and when just the slopes are equal we have GCA. The isoboles are extended in an intuitive way when the slopes vary beyond $\beta = 1$.

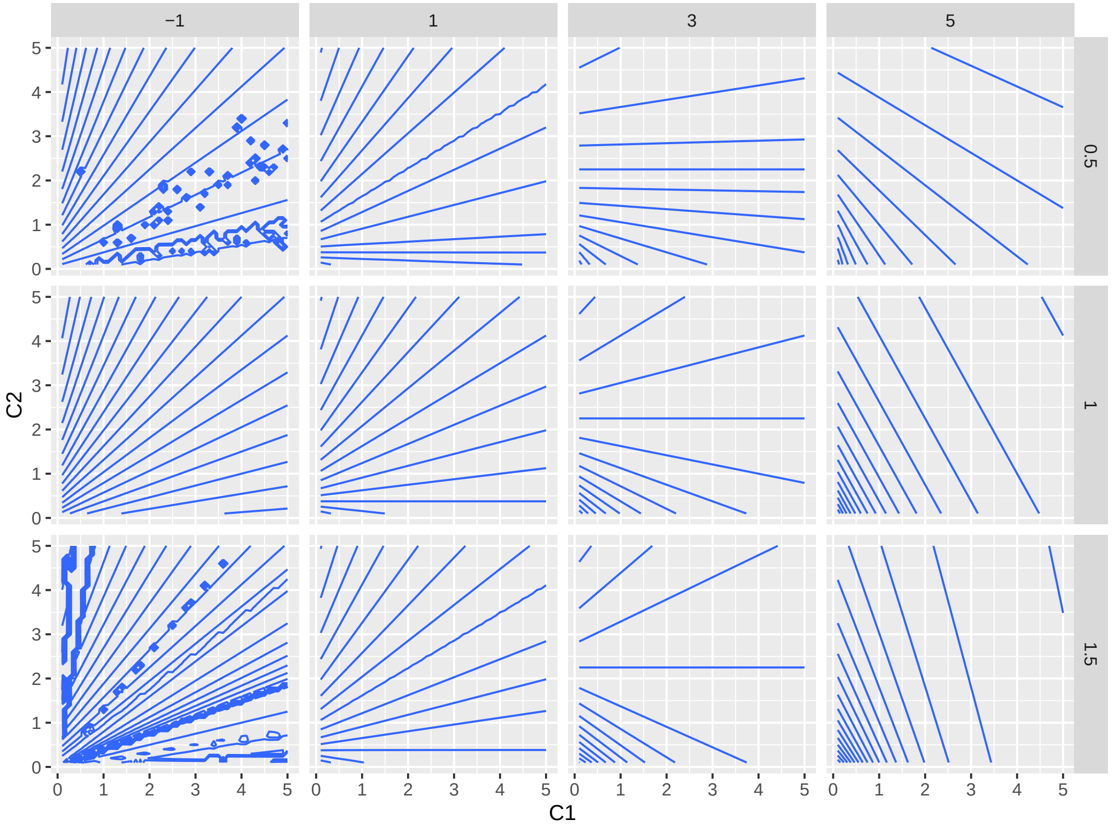

Figure 1: **Isobole plots for various parameters.** Isobole plots showing lines of equal response for a mixture of two chemicals with various parameter settings. The top row shows the sill for chemical 1 (c1), where the other chemical has a sill of 5. The right side shows the slope parameter, while the other chemical has a slope of 1. The center plot in the rightmost column is CA, the center row is GCA, and the lower and upper rows show how our reflection method extends GCA for non-unit slopes when using the Hill function. The anomalies in the first column are due to the negative sill, which leads to multiple solutions (slope<1) or no solutions (slope>1) in GCA.

1.3 Proof Sketch, existence of GCA solution with positive sills

The GCA Eq 2 consists of concentrations c_i with coefficients $1/f_i^{-1}(R)$. When plugging in positive values for R, the coefficients have range $[-1/(2\theta_i), \infty)$, where $R \to \infty$ results in $-1/(2\theta_i)$ and $R \to 0$ leads to a coefficient of ∞. This can be seen by first checking that the inverse function $f_i^{-1}(R)$ will have range $[-\infty, -2\theta_i] \cap [0, \infty]$ for $R > 0$. This means that, for very large R, all of the concentrations get a negative coefficient, while decreasing R will continuously and monotonically increase all coefficients to positive infinity. This means the

left side of Eq 2 will continuously vary from a negative value to positive infinity. Hence there exists (by continuity) a unique (by monotonicity) value for R that will yield a sum of 1.

When negative sills are present, the uniqueness is no longer guaranteed. As illustrated in Fig 2, depending on the magnitude of the negative sill and slope, the monotonicity of the combined coefficients can be disrupted in the form of a dip or bump that results in up to two additional possible solutions that solve Eq 2.

2 Bayesian Model Specification

For our Bayesian hierarchical model, we use weakly informative priors to provide some penalization of extreme parameter values while avoiding the issues of noninformative uniform priors, which can either lead to maximum likelihood fits with extreme values or can be sensitive to boundaries [1].

2.1 Hierarchical Model, Random Effects

$$R_i(c) = f_i(c|a_i, \theta_i, \beta_i, u_{ij}, v_{ij}) = \frac{a_i + u_{ij}}{1 + \left(\frac{\theta_i}{c}\right)^{\beta_i}} + v_{ij} + \epsilon_{ijc}$$

$$a_i \sim N(0, 200), \quad \beta_i \sim G(1, 1), \quad \theta_i \sim G(0.01, 0.01)$$

$$u_{ij} \sim N(0, \sigma_u), \quad v_{ij} \sim N(0, \sigma_v), \quad \epsilon_{ijc} \sim N(0, \sigma)$$

For identifiability, we fix $u_{1j} = v_{1j} = 0$ for the first replicate of each chemical. Weakly informative conjugate inverse gamma priors are chosen for $\sigma_u, \sigma_v, \sigma$, with hyperparameters chosen to yield a high variance: $IG(0.1, 0.1)$.

A Gibbs sampling procedure can be used for the effects a_i, u_{ij}, v_{ij} and variance terms $\sigma_u, \sigma_v, \sigma$ because they have conjugate priors and closed-form posteriors. The remaining terms θ and β occur as nonlinear elements and are sampled via Metropolis-Hastings. The slope term β has proposals generated from truncated normal distributions centered at the previous value. The possible values for the EC_{50}parameter covers multiple orders of magnitude, so the proposals are generated following a lognormal distribution: a small Gaussian step is made from the log of the current estimate, and then that proposal is exponentiated to provide a new candidate parameter.

2.2 MCMC convergence

We perform a Gelman-Rubin tests of convergence and mixing to determine if our model fitting procedure is actually becoming stationary or mixing poorly. The test statistic assumes a Gaussian posterior, which requires a log-transform for some chemicals. The results are in table 1 and suggest that most of the chains converge reasonably well. Some chemicals like chemical 17, benzyl butyl phthalate, mix poorly as a result of identifiability issues described in Figure 2 and in Section 3.2.

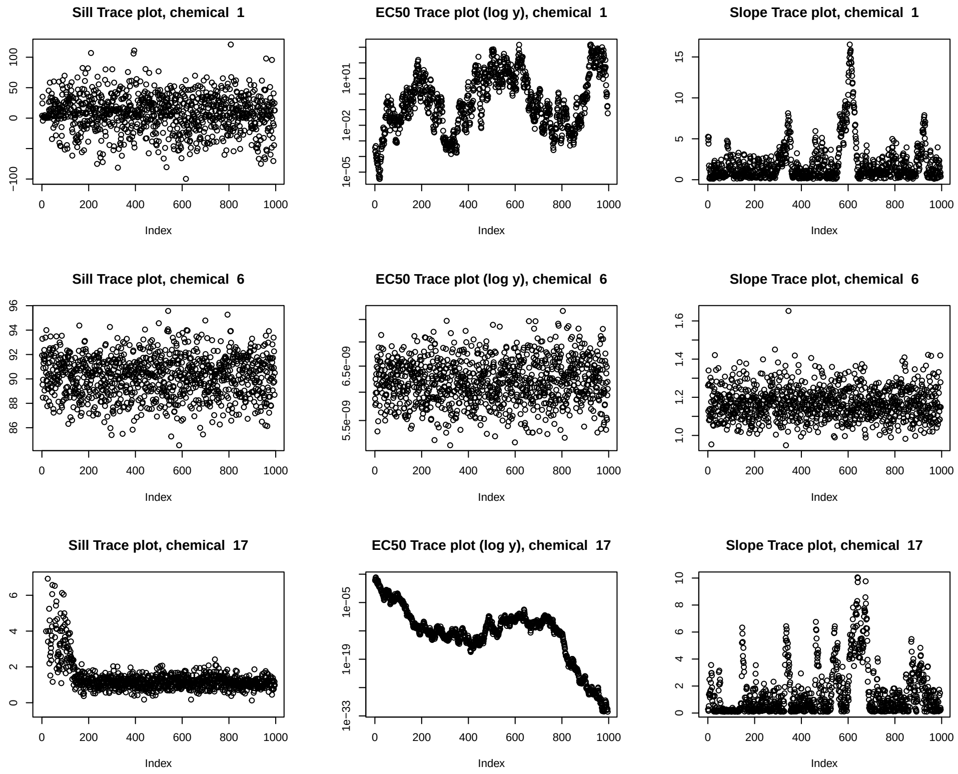

Figure 2: **MCMC stationarity.** MCMC trace plots for the sill, E50, and slope parameters show samples drawn from the posterior after a burn-in of 5000 iterations and thinning the remaining 20,000 iterations down to 5000 points. The $EC_{50}$for chemical 17 (benzyl butyl phthalate) was the only trace that failed to converge to a stationary distribution according to the Gelman-Rubin diagonostic, but other parameters such as the $EC_{50}$for chemical 1 do not have ideal trace plots. Failures to reach a stationary state for some chemicals and parameters suggests an identifiability issue where there is no actual response in the data.

CAS	Name	Sill		EC_{50}		Slope		Sigma		U RE Variance	
		Point	Up CI	Point	Up CI	Point	Up CI	Point	Up CI	Point	Up CI
107-15-3	Ethylenediamine	1.00	1.01	1.05	1.16	1.11	1.30	1	1.00	1.00	1.01
143-50-0	Kepone	1.08	1.23	1.06	1.17	1.02	1.04	1	1.00	1.04	1.12
15972-60-8	Alachlor	1.03	1.08	1.03	1.09	1.02	1.04	1	1.00	1.01	1.03
17924-92-4	Zearalenone	1.01	1.04	1.01	1.02	1.00	1.01	1	1.00	1.00	1.00
34256-82-1	Acetochlor	1.02	1.05	1.02	1.05	1.01	1.02	1	1.00	1.01	1.03
434-07-1	Oxymetholone	1.00	1.00	1.00	1.00	1.00	1.00	1	1.00	1.00	1.00
50-02-2	Dexamethasone	1.00	1.00	1.00	1.00	1.00	1.00	1	1.00	1.00	1.00
50-29-3	p,p'-DDT	1.02	1.07	1.04	1.11	1.27	1.92	1	1.00	1.00	1.01
52806-53-8	Hydroxyflutamide	1.00	1.00	1.00	1.00	1.00	1.01	1	1.00	1.00	1.00
57-83-0	Progesterone	1.00	1.00	1.00	1.00	1.00	1.00	1	1.00	1.00	1.00
63-05-8	4-Androstene-3,17-dione	1.00	1.00	1.00	1.00	1.00	1.00	1	1.00	1.00	1.00
71-58-9	Medroxyprogesterone acetate	1.00	1.00	1.00	1.00	1.00	1.00	1	1.00	1.00	1.00
76-43-7	Fluoxymestrone	1.01	1.02	1.01	1.03	1.00	1.01	1	1.00	1.01	1.04
80-05-7	Bisphenol A	1.00	1.01	1.01	1.01	1.13	1.21	1	1.00	1.00	1.00
80-43-3	Dicumyl peroxide	1.02	1.08	1.22	1.62	1.10	1.32	1	1.01	1.07	1.21
84852-15-3	4-Nonylphenol, branched	1.02	1.04	1.02	1.04	1.01	1.02	1	1.00	1.00	1.01
85-68-7	Benzyl butyl phthalate	1.07	1.18	3.09	7.00	1.10	1.19	1	1.00	1.00	1.01
90-05-1	2-Methoxyphenol	1.03	1.10	1.27	1.80	1.17	1.30	1	1.00	1.00	1.01

Table 1: **Tests for MCMC stationarity.** Gelman-Rubin Diagnostic statistics for the MCMC trace plots of the model parameters of interest. Values near 1 suggest convergence to a stationary distribution, while values above 2 suggest a failure to reach stationarity. Values computed with three chains of 25,000 iterations each. Other parameters showed diagnostic values near 1 and are not included to be concise

3 Data Tables

CAS	Name	Sill	EC_{50}	RE SD	Int SD	Slope
107-15-3	Ethylenediamine	8.91	$2.55e-01$	3.34	0.44	1.12
143-50-0	Kepone	– 18.10	$1.16e-04$	9.64	0.86	1.60
15972-60-8	Alachlor	– 38.42	$4.98e-04$	11.89	0.56	0.81
17924-92-4	Zearalenone	– 30.38	$3.26e-04$	1.41	0.54	0.70
34256-82-1	Acetochlor	– 27.35	$2.83e-04$	12.69	1.62	0.85
434-07-1	Oxymetholone	90.34	$6.20e-09$	3.29	4.96	1.16
50-02-2	Dexamethasone	357.85	$5.52e-09$	6.61	35.33	1.05
50-29-3	p,p'-DDT	– 25.05	$1.07e-04$	1.47	0.74	4.94
52806-53-8	Hydroxyflutamide	33.58	$4.01e-06$	15.68	0.65	1.83
57-83-0	Progesterone	96.79	$1.45e-06$	2.13	1.05	0.88
63-05-8	4-Androstene-3,17-dione	109.96	$3.19e-08$	3.97	1.37	0.89
71-58-9	Medroxyprogesterone acetate	238.26	$7.87e-09$	3.65	55.55	0.59
76-43-7	Fluoxymestrone	152.85	$7.74e-09$	57.20	4.96	0.17
80-05-7	Bisphenol A	– 17.06	$5.67e-04$	2.19	0.60	0.55
80-43-3	Dicumyl peroxide	– 10.72	$3.17e-03$	7.86	1.03	0.51
84852-15-3	4-Nonylphenol, branched	– 31.41	$2.05e-03$	19.53	0.58	0.52
85-68-7	Benzyl butyl phthalate	1.17	$2.08e-12$	1.25	1.01	0.78
90-05-1	2-Methoxyphenol	20.11	$8.27e-02$	1.61	0.45	0.34

Table 2: **Full posterior summary statistics.** Parameter estimates from the MCMC RE model, computed as the posterior median. The values are rounded to 2 digits or 3 significant digits depending on scale. RE SD refers to the Sill random effect standard deviation and Int SD refers to the intercept random effect standard deviation (the intercept is assumed to be 0). Poster variance estimates are available for all parameters on request.

Mix	MSE						
	RGCA	RGCA Sill	RGCA KM	RGCA Rand	GCA	IA	CA
1	205701	74784	9807	11097	160824	1504	154712
5	14839	15639	9509	4058	24437	4043	4204
10	11616	11721	25234	30766	9782	56703	80622
12	283131	26076	18412	14532	158166	6419	116386
20	181657	31661	6728	5968	146457	4007	146606
25	30571	30350	6351	10143	30430	23530	35573
29	53287	8374	7634	8591	29808	13436	152417
30	30483	30298	13628	18205	30512	33739	55509
31	6456	6696	8222	14196	11009	20214	21215
32	14329	15204	10611	8313	19575	10150	8841
43	129906	43245	3276	5360	124152	3194	223697
45	11101	11266	6418	12459	11504	21871	24509
50	7932	8177	4155	6512	6910	13562	26744
52	304343	38938	27816	19491	130669	9882	79255
55	224073	99881	17898	23900	194814	5069	157885
57	198597	22045	13292	12317	147096	7429	119138
62	145652	13485	3818	3063	58548	8510	174757
Best	1	0	5	2	1	8	0

Table 3: **MSE scores for Tox21.** Mean squared error values for the Tox21 AR-luc mixture predictions using the methods described in the main text. RGCA uses no clustering, RGCA Sill uses the postive sills as one cluster and all other sills as another cluster, and RGCA KM refers to K-means clustering, RGCA Rand refers to random clustering.

Mix	CRPS						
	RGCA	RGCA Sill	RGCA KM	RGCA Rand	GCA	IA	CA
1	9173	6378	2578	2019	9854	940	9168
5	2758	2803	2044	1156	4005	1661	1677
10	2513	2532	2929	3752	2061	6156	7590
12	9497	3614	2953	2307	10244	1892	7169
20	8691	4315	1822	1504	9741	1573	9394
25	3810	3803	1738	2113	3549	4218	5202
29	4102	1829	1444	1502	4515	2402	11340
30	3741	3747	2295	2736	3567	4777	6214
31	1828	1872	1953	2514	2232	3926	4076
32	2431	2508	2048	1838	3045	2549	2413
43	6758	4325	1378	1218	8001	1445	12414
45	2366	2399	1629	2309	2272	4132	4515
50	2041	2074	1318	1622	1977	3206	4666
52	10323	4842	4070	2938	9871	2444	6088
55	9972	7429	3271	3110	10760	1762	9368
57	8955	3414	2466	2104	10207	2074	8344
62	6178	2577	1469	1143	5605	2470	10804
Best	1	0	5	5	1	5	0

Table 4: **CRPS scores for Tox21.** Continuous rank probability scores (CRPS) for the Tox21 AR-luc mixture predictions using the methods described in the main text. RGCA uses no clustering, RGCA Sill uses the postive sills as one cluster and all other sills as another cluster, and RGCA KM refers to K-means clustering, RGCA Rand refers to random clustering.

Mix	LLH						
	RGCA	RGCA Sill	RGCA KM	RGCA Rand	GCA	IA	CA
1	1205.0	*Inf*	349.4	319.7	*Inf*	*Inf*	*Inf*
5	5143.1	3482	1097.5	342.5	*Inf*	*Inf*	*Inf*
10	*Inf*	*Inf*	574.6	828.6	*Inf*	*Inf*	*Inf*
12	564.8	5550	1626.7	407.2	*Inf*	*Inf*	*Inf*
20	663.3	7463	334.8	310.9	*Inf*	*Inf*	*Inf*
25	*Inf*	*Inf*	412.3	458.7	*Inf*	*Inf*	*Inf*
29	391.5	1029	309.3	308.7	*Inf*	*Inf*	*Inf*
30	*Inf*	*Inf*	452.7	529.7	*Inf*	*Inf*	*Inf*
31	1496.5	1335	552.3	535.3	*Inf*	*Inf*	*Inf*
32	*Inf*	*Inf*	1203.8	634.7	*Inf*	*Inf*	*Inf*
43	1351.6	*Inf*	286.1	283.3	*Inf*	*Inf*	*Inf*
45	3611.1	2806	409.6	447.4	*Inf*	*Inf*	*Inf*
50	2185.2	2209	445.1	466.2	*Inf*	*Inf*	*Inf*
52	659.3	*Inf*	2960.4	585.0	*Inf*	*Inf*	*Inf*
55	2225.6	*Inf*	496.7	459.7	*Inf*	*Inf*	*Inf*
57	563.3	4059	597.6	385.2	*Inf*	*Inf*	*Inf*
62	414.0	4448	338.3	310.8	*Inf*	*Inf*	*Inf*

Table 5: **LLH scores for Tox21.** Log likelihood scores for the Tox21 AR-luc mixture predictions using the methods described in the main text. RGCA uses no clustering, RGCA Sill uses the postive sills as one cluster and all other sills as another cluster, RGCA KM refers to K-means clustering, and RGCA Rand refers to random clustering. GCA, IA, and CA are predicted without uncertainty and have infinite LLH.

Mix	CAS	Description
1	NOCAS_49550	Graded concentrations with androstenedione and oxymetholone driving the response
5	NOCAS_49509	Graded concentrations with dexamethazone and medroxyprogesteron acetate driving the response
10	NOCAS_49511	Graded concentrations with o-methoxyphenol and hydroxyflutamide driving the response
12	NOCAS_49545	Graded concentrations with progesterone and dexamethozone driving the response with ER agonists at 4x EC50 response
20	NOCAS_49543	Graded concentrations with oxymetholone and fluoxmetrone driving the response with ER agonists at 4x EC50 response
25	NOCAS_49513	Graded concentrations with androstenedione and oxymetholone driving the response
29	NOCAS_49538	EQUICONCENTRATION SOLUTIONS
30	NOCAS_49512	Graded concentrations with hydroxyflutamide and androstenedione driving the response
31	NOCAS_49507	Graded concentrations with fluoxymeterone and progesterone driving the response
32	NOCAS_49508	Graded concentrations with progesterone and dexamethozone driving the response
43	NOCAS_49548	Graded concentrations with o-methoxyphenol and hydroxyflutamide driving the response with ER agonists at 4x EC50 response
45	NOCAS_49506	Graded concentrations with oxymetholone and fluoxmetrone driving the response
50	NOCAS_49510	Graded concentrations with medroxyprogesterone acetate and o-methoxyphenol driving the response
52	NOCAS_49546	Graded concentrations with dexamethazone and medroxyprogesteron acetate driving the response with ER agonists at 4x EC50 response
55	NOCAS_49549	Graded concentrations with hydroxyflutamide and androstenedione driving the response with ER agonists at 4x EC50 response
57	NOCAS_49544	Graded concentrations with fluoxymeterone and progesterone driving the response with ER agonists at 4x EC50 response
62	NOCAS_49547	Graded concentrations with medroxyprogesterone acetate and o-methoxyphenol driving the response with ER agonists at 4x EC50 response

Table 6: **Tox21 Mixture Descriptions** Includes placeholder CAS numbers used in plots for reference.

4 Individual Curve Fits

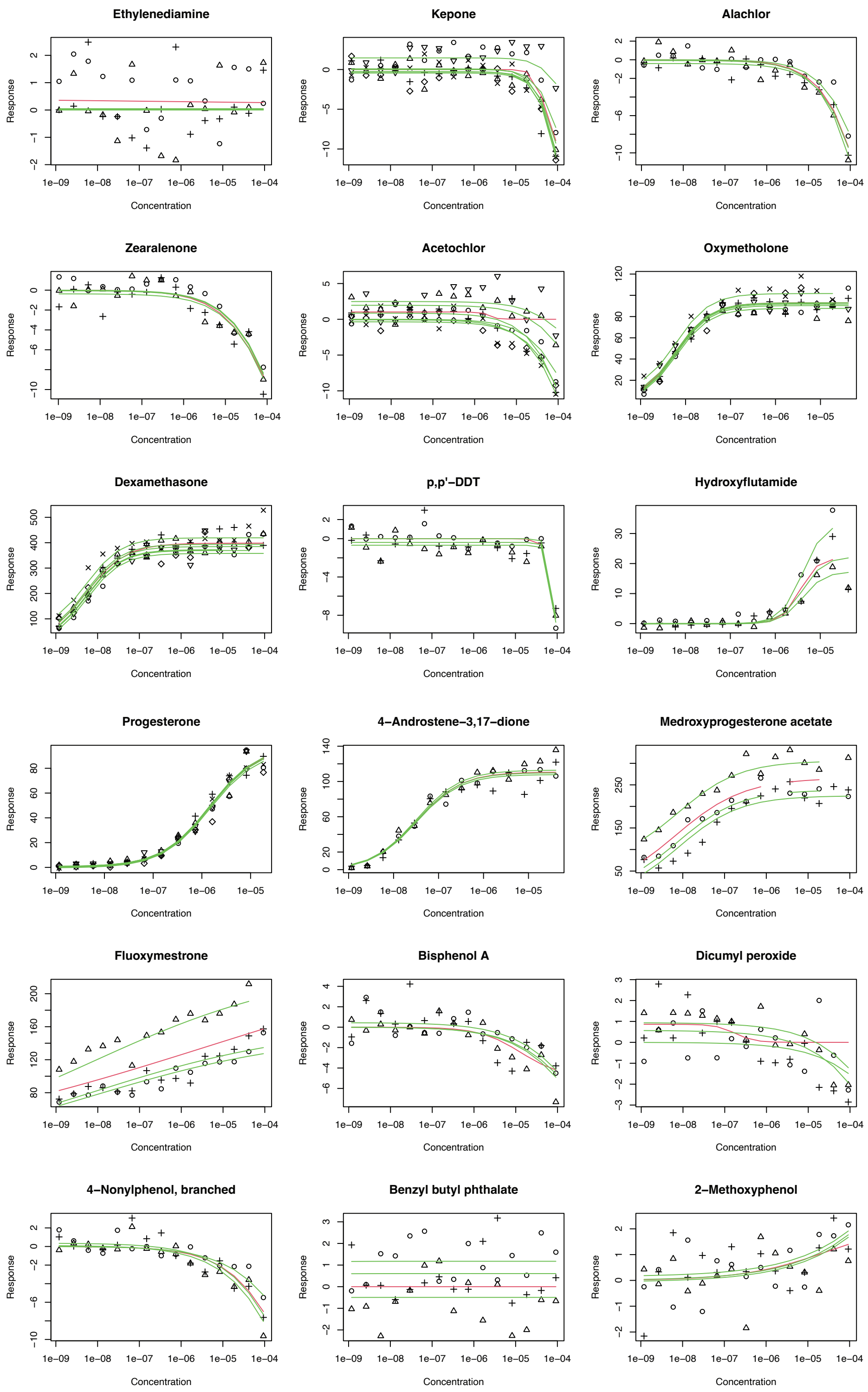

Figure 3: **Individual dose response curves plotted with replicates.** The red line is the resulting curve from the `drc` package while the green curves represent our model fits with one curve per replicate.

5 Mixture Predictions

In this section we show a few representative examples of dose response prediction. For the full mixtures, our method often shows good coverage of the observed data, but sometimes IA or GCA would better predict the mean effect. Some smaller mixtures were not predicted correctly by any method due to synergy or antagonism. However, our method has the capability to consider multiple clusterings and in some cases generates credible intervals that are wide enough to cover the observed data.

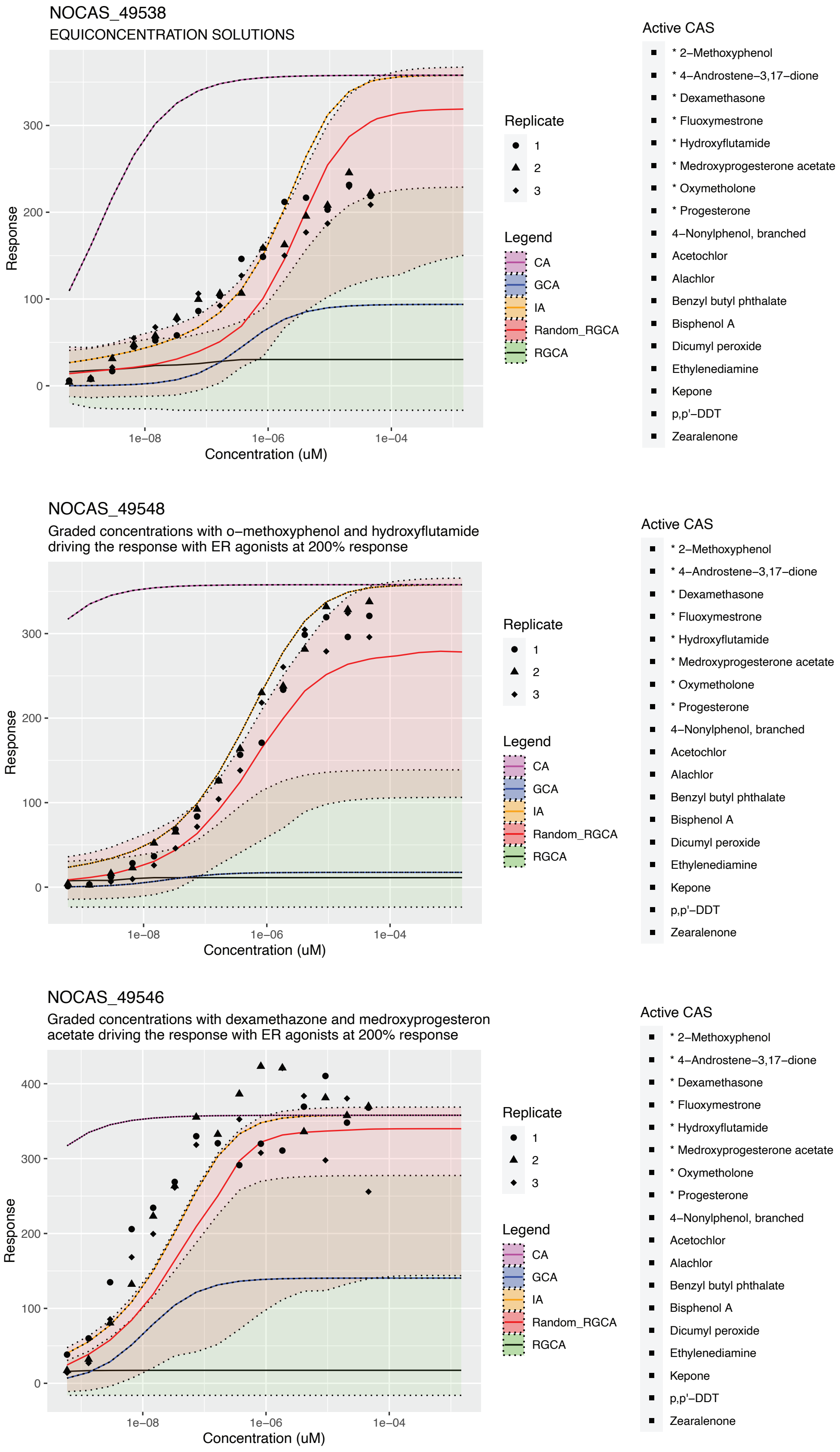

Figure 4: **Examples of mixture dose response curves plotted with replicates.**

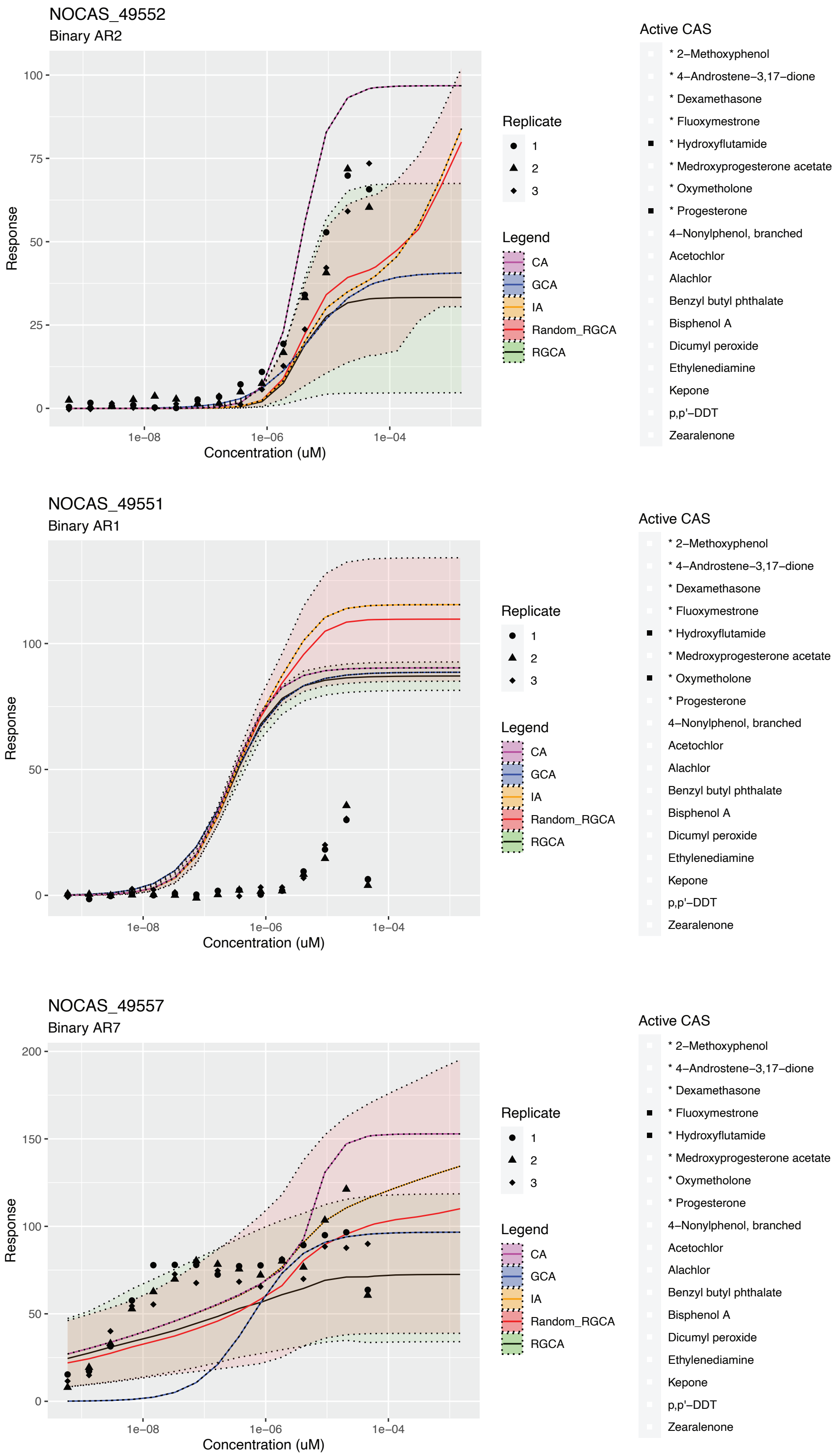

Figure 5: **Examples of binary mixture dose response curves plotted with replicates.** In most cases, all of the predictions are incorrect due to antagonism or synergy. In a few cases, the uncertainty from considering different clusterings is large enough to cover the observations.

References

[1] Wheeler MW. An investigation of non-informative priors for Bayesian dose-response modeling. Regulatory Toxicology and Pharmacology. 2023;141:105389.
