# Supplementary material for: Reflected generalized concentration addition and Bayesian hierarchical models to improve chemical mixture prediction
Source: PLoS One. 2024 Mar 28;19(3):e0298687. doi: 10.1371/journal.pone.0298687 (PMC10977799; doi:10.1371/journal.pone.0298687)
Supplement: Attachment — Submitted filename: Response_to_Reviewers.pdf [file pone.0298687.s002.pdf]

[We'd like to thank the editors and reviewers for taking the time to read and comment on our manuscript. The feedback was very helpful and we feel the current iteration of the manuscript is a great improvement over the initial submission. We have made substantial changes to the presentation, including a significantly expanded section describing the mathematical details of our approach and a new plot that provides insight into the consequences of our modeling choices. We hope the reviewers and editors agree that the revision has improved the quality of the manuscript and that it is worthy of publication.]

Reviewer 1: The development of joint toxicity modeling has been one of the challenges in the field, to which the authors of this work have made their own contribution. However, some issues still require further explanation or expansion of the data by the authors.

1. Lines 21-22: GCA is proposed to compensate for the shortcomings of the CA model, so it is suggested that the authors also need to provide the predictions of the CA model for comparison.

   [Concentration Addition is not readily applicable in the case of partial agonists, where different chemicals have different maximum responses. We have attempted to include CA by rescaling all sills to 1 and then scaling the response back to the maximum sill fit to the components, but this is not the traditional implementation and only serves to show the potentially enormous error.]

2. Lines 24-25: The presentation is too general and the predictive power of the developed models should be described quantitatively. For example, how many times higher than the predictive power of traditional models?

   [We include a number of metrics to quantitatively compare the measurements across multiple mixtures, including a proper scoring rule, the CRPS, that has the guarantee that the most correct method will give the highest score. Since the total error is not expected to scale linearly with the parameters and mixture size, it is difficult to provide a general percentage improvement that can be expected. The error may also depend on unknowns such as the presence of synergy.]

3. Lines 28-29: This problem is difficult to solve; after all, the models developed are also based on the prerequisite of non-interaction between the mixed components. And, existing models require a clear mode of toxic action.

   [This is indeed the case. Our intention is illustrate how one might go about making a prediction with only minimal knowledge of mode of action and the language has been adjusted to clarify this point.]

4. 2.1 section: Personally, I think that there is no theoretical basis for the integration of these two models into one. the GCA model is developed on the basis of the CA model on the premise that the mixed components should have the same modes of toxic action, whereas the IA model is applied on the premise that the mixed components have different modes of toxic action.

   [This is correct, in the sense that IA and GCA represent different, non-equivalent ways to combine toxic responses. The premise of the two-step approach is to leverage this difference by treating a group of chemicals with a similar mode of action as a single pseudo-chemical with a carefully chosen dose (ie the GCA step). If there are multiple modes of action that are suspected to be used across the chemicals, we have multiple pseudo-chemicals that represent different modes of action that will need to be further aggregated by IA to get a single dose response.]

5. Figure 6: Too many constraints may limit the predictive efficiency of the model.

   [We did not understand this comment.]

6. 2.3 section: The incorporation of Bayesian Data Model may reduce the predictive efficiency of the model.

   [We presume that the reviewer is referring to the potential influence of the prior, which can introduce bias compared to frequentist approaches which are often unbiased or based on maximum likelihood. However, in the context of dose response modeling, a maximum likelihood approach can give unreasonable estimates, as illustrated by the drc package. Furthermore, the Bayesian approach offers posterior uncertainty that is used for estimating credible intervals, which are critical for uncertainty estimation when posterior (or MLE) distributions are not Gaussian.]

7. Result section: Increasing the results of the developed model in comparison with the CA model is more convincing.

   [We have included an interpretation of CA for comparison. However, CA assumes all sills are equal and does not apply to partial agonists, which we have. The predictive performance is low.]

8. Lines 427-441: In addition to considering interactions between hybrid components, the authors need to consider applying additional data sets to validate the models developed. In addition, the model involves too many parameters, which requires a larger amount of data for validation. From a risk assessment perspective, the practical applicability of the model still requires further consideration.

[While there are many parameters in total, each chemical is just fit with a three-parameter Hill model, which does not require large data sets the way machine learning methods do. We feel that testing with 17 different mixtures in the Tox21 data set is a large amount of validation data, given that many papers only compare to one mixture. We agree that the risk assessment is inadequate for use in policy making and requires more development, but in cases where there is no clear understanding of the danger of a mixture, we feel our method offers a convenient baseline solution.]

In summary, the authors of this study are still working hard to try to advance the mixture toxicity model. After considering the above reviewer' comments, the work is worthy of publication.

[Thank you for your review!]

Reviewer 2: The manuscript refers to a specific mixture scenario where mixture components have differences in efficacy (ie, dose-response curve maxima) and, as consequence, the calculation of an expected mixture effect according to dose additivity is restricted. Solutions for this problem have been published previously but rely on narrow, usually unrealistic model assumptions (Howard &Webster) or provide a range of worst-case mixture predictions (Toxic unit extrapolation, Scholze et al). Here the manuscript suggests a mathematically-motivated more flexible approach. This is integrated in a chemical grouping approach where the authors make the assumption that the model parameter describing the steepness of a dose-response relationship is capable of showing a toxicological mode of action. As much as I would like to see the use of novel mathematical & statistic methods in mixture toxicology, I have serious problems with the authors' methods as they rest on assumptions which are not in line with current knowledge in mixture toxicology and pharmacology:

The steepness of dose-response pattern is not indicative for the pharmacological/toxicological mode of action, a stubborn myth which experimental and theoretical toxicology have disproved now for decades. Compounds with nearly identical hazard profiles but different kinetic properties can result into data of very different dose-response shapes, compounds with different targets can produce nearly identical dose-response pattern, and this holds true for in vivo and in vitro study endpoints. So no, establishing cumulative mixture assessment groups according to the steepness of dose-response patterns is not an option anybody would suggest, and I cannot think about any application where a grouping of this specific model parameter under consideration of statistical variability would be useful.

[We have adjusted our narrative to satisfy this fact. While our total method still seems to outperform CA and GCA, we agree that our justification of clustering slope as a proxy for mode of action is not supported in the literature. After investigation spurred by questions from the other reviewers, we found that random clustering provides a similar improvement, as does clustering on the sill rather than the slope. The sill parameter is arguably easier to justify, since there is a reasonable starting point: chemicals with a positive sill share one mode of action and chemicals with 0 sill share a different mode of action (ie no effect). The message of our method is that the mixtures are probably not independent, and the random clustering can help cover all possible outcomes.]

A receptor-based bioassay endpoint from eg the AR-luc assay is not capable of showing responses to more than one "mode of action". In fact, most cell-based assay endpoints from the ToxCast library are annotated as MIE's, and therefore considered as ideal for being in line with the pharmacological assumptions of Concentration addition. Also, factors like (i) nominal vs bioavailable (or target) concentrations, (ii) cytotoxicity at high concentrations,

and (iii) lack of data support for weak assay responses and their impact on model complexity, can impact the curve estimate from an empirical dose-response model.

[The assumption that there must be one mode of action and therefore CA or GCA should predict the mixture response is surprisingly not supported. As we describe in the results, IA often offers a better prediction that GCA or CA for larger mixtures. This could be explained by the factors suggested, such as bioavailability or cytotoxicity. For individual chemicals, cytotoxicity was flagged in the data and removed before analysis to help avoid that influence. Any issues with bioavailability are assumed to be present in both individual and mixture data and therefore don't need to be accounted for. If bioavailability depends on the mixture, this is a synergistic or antagonistic effect which we wouldn't be able to model anyway. It is possible that all of the effects we are predicting are the result of synergy and GCA, and our predictions work just by coincidence, but it is beyond the scope of our work to determine if this is the case.]

Demonstrating by simulation that IA and CGA cannot describe a mixture scenario accurately that doesn't fulfil the conceptual requirements of these two mixture models is trivial, so I don't understand the meaning of paragraph 2.6, especially as it is promised to be a "validation".

[The triviality of the validation is intentional. One would assume that a mixture model that is based on a two step model cannot be estimated with a one-step model, but a skeptic could argue that the difference in predictions would be insignificant and that either one-step model should be used. We have added additional simulation comparisons to illustrate how the methods compare, using one method as the truth and and the other models for predicting. By comparing so many methods, a reader can gain some intuition such as "the difference between a two-step RGCA and GCA when the two-step is true is about half the difference between GCA and IA when GCA is true". It is not perfect, but we thought it is the most intuitive way to compare so many methods that predict functions (rather than points).]

- Dose-response data from the AR-luc assay were used for 18 compounds, with 7 positively identified as AR antagonists and the remaining 11 known to be ER agonists. The authors say "there are 69 different mixtures" but without explaining the "where", and it is unclear why and how these data sets were used for the simulations. For this endpoint only C(G)A is a reasonable mixture reference, and this has been demonstrated in numerous mixture studies. So why considering IA and RGCA+DP(I assume this refers to the proposed CGA/IA model)?

  [We have added additional details about the mixtures we considered. We agree that

GCA and CA are reasonable mixture references but the predictive performance is low. We think it is reasonable to consider grouping the ER agonists and AR agonists separately, and the results are better. Hence we follow the train of thought that there are unknowns in the mode of action and propose to try other groupings to see if the performance improves. We have clarified our narrative based on this idea: there is uncertainty in the mode of action, and the two step procedure can improve estimates.]

- Real data from experimental mixture studies are not used (or shown) to assess the approach, so I cannot see evidence in favour for this method.

  [The Tox21 data set has real experimental results for individual and mixture data. We are not aware of a "more realistic" source of data.]

- I cannot see how the extension of the CGA model by Hobster & Webster works, as Eq 6 refers only to $\beta = 1$, ie the regression model used by Hobster&Webster. Where is the novelty?

  [Equation 6 had a typo that has been corrected. RGCA is defined for $\beta > 0$ and reduces to GCA for the case of $\beta = 1$.]

- My recommendations:

  - The extension of the CGA model by Hobster&Webster deserves certainly its own publication, but here I would expect a better and more clear mathematical presentation, the (explicit or implicit) mathematical equations for the case $\beta! = 1$ (Eq 6 refers only to $\beta = 1$, or?), statements about the model domain (what are the limitations?), and a real mixture scenario to demonstrate its applicability (as in Howard&Webster or Scholze et al). The reader must be in the position not only to use the method but also to repeat the calculation for any example shown.

    [Additional detail has been added, see section 2.2.]

  - If I got it right, the Bayesian aspect refers mainly to the between-study variability in the dose-response modelling, which is not novel (eg, the recommended approach by EFSA/Europe in the derivation of a regulatory benchmark dose). Here I would suggest to expand this to the proposed CGA model, expressing the statistical uncertainty of CGA prediction via a credibility interval is certainly something of great interest in the mixture field.

    [The methodology we propose is Bayesian in that we estimate full posterior distributions for all parameters. The uncertainty is then based on the data provided for each individual chemical and the choice of priors, rather than between-study

variability. The secondary clustering step also introduces uncertainty that we combine with parameter uncertainty through our sampling scheme. We agree that the procedure can be applied equally to GCA. To contrast with a method that has uncertainty quantification, we show GCA and CA predictions using direct parameter estimates, without the benefit of the Bayesian credible intervals but with the benefit of the Bayesian parameter estimation procedure. In other words, the curves are still centered according to what the Bayesian version would produce. We present our methods with the credible intervals, showing the posterior uncertainty for individual chemical fits may or may not cover the observed mixture response. We further argue that large errors imply that something is missing from the GCA model that is at least partially recovered by considering the two-step approach.]

– I would also strongly recommend to align with a mixture toxicologist.

  [Before submission, this work was presented to a group with multiple mixture toxicologists and the assumption that slope can inform the mode of action was treated as plausible but uncommon.]

[Thank you for your review!]

Reviewer 3: In their manuscript "Reflected Concentration Addition to Improve Chemical Mixture Prediction" Daniel Zilber and Kyle Messier suggest an adopted approach to predict effects of chemical mixtures from observed effects of the mixture components.

In my view the approach is very interesting and plausible and can be a very valuable contribution for the field of mixture toxicology. The manuscript suffers in part from some ambiguities (see below) which should be addressed before publication. Overall, I would recommend considering the manuscript for publication after moderate revisions.

In my view, the following major topics should be addressed in a revision:

1. Incomplete and biphasic concentration responses in Tox21 Data:

   - An accurate mixture prediction depends a lot on accurate concentration response models for the mixture components. The authors partly address this issue by integrating a set of noise terms in the Hill function used. Yet, it did not become clear to me, how the authors deal with a) biphasic responses (e.g. due to cytotox) as it can be seen for progesterone or hydroxyflutamide in Figure 2. I guess the highest 2 (?) concentrations are just not considered for the model, but it is not mentioned anywhere. It might also have a large effect on the overall outcome which concentrations are selected for modeling. There are some studies on adopted CA for biphasic models (e.g. http://www.nature.com/articles/srep17200) and it would be interesting to see how these compare to the proposed RGCA model. Since this could be quite a lot of additional work and thus not feasible, I think it would be really good to include this topic in the discussion.

     [Such drastic drops in response were indeed flagged as cytotoxicity and we removed any flagged data points before analysis. We have included this detail in the manuscript and added a comment about biphasic responses to the discussion as a limitation to our method.]

   - b) "incomplete" concentration response/unknown maximum effect: The authors thoroughly address the topic of partial agonists/variable maximum effect. Especially for High-Throughout-Data like Tox21, we regularly have the issue, that effects do not reach a plateau, so we only have an incomplete curve and a maximum effect cannot be identified. It gets even worse for the case of cytotox, where the maximum effect is even more obscured. This does not matter for calculating Benchmark Concentrations, but is a very important limitation in mixture modeling. It would be very helpful if the authors make more explicit how the maximum effect is modelled. If feasible, they could make a very strong point, when they

could test the sensitivity of different alpha ranges on mixture prediction. I am aware, that this might be too much outside the scope of the study, but it should made explicit in the methods and included as limitation in the discussion at least. *[The maximum effect was accounted for through our Bayesian model, which is one of the advantages of a Bayesian approach compared to a maximum likelihood approach. A principle of Bayesian statistics is to provide a prior belief or distribution of what the maximum value could be, and allow the data to update this distribution. In comparison to a maximum likelihood approach, the estimates of the maximum effect given an incomplete curve will be much smaller and more reasonable for the Bayesian model. Also the posterior will provide uncertainty, allowing for smaller maximum effects to have some weight in the subsequent sampling and prediction steps.]*

2. 2) Benefits of the Bayesian Hierarchical Models in Mixture Modelling: As the authors point out, earlier mixture studies have shown that Observed Mixture Responses often end up somewhere between IA and CA. When reading the manuscript I wondered, if one could arrive at the same quality of prediction as with the Bayesian Clustering by just randomly assigning the clusters (maybe still based on slope, but e.g. a random cluster number). Would you dare to try it out? ;-)

   [We accept your dare! It turns out that the results are indeed comparable and have been included, but this isn't a bad thing. The key point we want to make is that quantifying the uncertainty in the parameters and treating the clustering as a version of noise can make a better prediction.]

3. I found the manuscript very interesting and inspiring to read, but I got the feeling that there should be some switching between SI and manuscript paragraphs. In some parts, established methods (like DPMM) were explained in detail - which is a nice service to the reader - but distracts a bit from the main adoptions and findings of this study. On the other hand, important information about the methods (e.g. how and why did we chose priors for single chemical modeling) are only implicitly mentioned or buried in the supplement. Maybe this is also a matter of taste, but I just want to encourage the authors to check theirs manuscript for this once more. Also make sure, that references to the SI are clear and easy to follow.

   [We have modified the manuscript to spend less time on the clustering details and more time on the RGCA component and the philosophy behind clustering. We included a few details about the priors but feel that the general audience will probably not appreciate the full specification, so we left it in the SI.]

Some more minor issues and questions in the following:

- **Abstract:** l. 13: "This is purely predictive..." This only becomes clear in the context of approaches that use mixture observations for modelling. Maybe reframe, e.g. "This predicts mixture responses based on observed responses of mixture components"...

  [The abstract has been rephrased for clarity.]

- l. 25: "significantly improved...": In my view, this does not go in line whith your discussion, where you state, that CRPS is your recommended metric for drawing conclusions (l. 389) and CRPS showed "marginally better" results (l. 386).

  [We had previously referred to figure 5b when showing significant improvements. 5b restricts the set of mixtures to those with all chemicals present at significant concentrations. Many of the mixtures were not predicted well by any method, which is what the overall marginal improvement reflects. However, we have reframed our analysis from the point of view of GCA and CA, which is what would typically be assumed with this data. We have tweaked the methodology and think the argument and results are more persuasive this time.]

- l. 28: "Lastly..." I would recommend to leave out this sentence, since this is not scope of the study

  [This line was removed.]

- **Introduction:** ll. 34ff: I share your concerns regarding "forever" chemicals, but was not convinced, that this group specifically adds to the mixture problem... *[Such chemicals are just an example of how we end up being exposed to mixtures and are not necessarily of primary concern over more active pollution from, say, transportation and industrial processes.]*

- ll. 39-51: I really liked this introduction into the world of mixture prediction! *[Thanks :)]*

- ll. 52ff: Maybe consider to cite the original publications for CA and IA? *[The original publications have been cited but one is in German and may not be very practical.]*

- l. 58: Could be misleading to talk about "adding" effects. Maybe reframe to "...so a series of chemical proportional effects can be multiplied..."?

  [It is true that IA can be written as a product, but it can also be written as a sum of scaled responses:

$$R = f_1(c_1) + (1 - f_1(c_1))f_2(c_2) + (1 - f_1)(1 - f_2)f_3(c_3) + \ldots = 1 - \prod(1 - f_i(c_i))$$

We want to preserve the additive intuition of both models but avoid spending a lot of time on IA, so we added an additional phrase to clarify that IA is addition of the responses while CA is addition of the doses.]

- ll. 60ff.: It took me a while to understand which probem(s) you are actually adressing with your study, maybe because there are these two elements of a) clustering similar mode-of-action and b) reflected gca, which both adress different types of problems. Maybe this could be made even more explicit in the introduction

  [We have adjusted the narrative of this manuscript to focus more on RGCA and to motivate its development in the context of two-step models. The two-step approach is not new and can be used for mixtures with multiple modes of action, but we argue that the existing techniques of CA and GCA require some compromise when fitting individual data, hence the need for RGCA.]

- l. 74: This statement did not became clear to me, maybe you could elaborate a bit more on this *[This statement was made more explicit.]*

- **Material and Methods:** “Algorithm 1”: This did not help me too much for understanding the approach. To be able to grasp this more intuitively, maybe a flowchart would be an option. Otherwise, it would at least help to explain/define all mentioned parameters and abbreviations, be a bit more consistent in the structure (e.g. “Step 1...Step 2... Step 3a...Step3b” in the “Result” and Approach). I could not find the referenced Equation 7 in the Supplement (Equations are not numbered there).

  [The algorithm has been updated and all relevant equations are now in the main document. Since algorithm is very linear and does not really involve branching decisions, we felt a flow chart would not help with clarity. We hope the current version is easier to understand.]

- Equation 3: Please also defineexplain parameters of this equation. How is alpha-max different from alpha (equation 1)? How do you deal with different alpha-max values in the IA part?

  [We have added a sentence to explain this parameter, which is defined for the entire mixture and does not change for each cluster.]

- Equation 6: I got a bit confused here. You are talking about a general form of inverse for beta not 1, but in Equation 6 you state beta = 1?

  [This is a typo that has been corrected.]

- l. 162: “iid Gaussian noise” - Typo?

[This is not a typo. iid is an abbreviation for "independent and identically distributed". Since this may confuse readers, we have written out the full phrase.]

- l. 170: Please state and justify all priors here or in the supplement. *[We state the priors for the three main parameters of interest along with justification but left the hyperpriors and other MCMC sampling details to the SI to avoid making a distraction.]*
- l. 180: Please explain how the data was filtered before modeling (see above).

  [We have added details in the data section describing how the data set has flags for values that are presumed to reflect cytotoxicity, and we use those flags to filter the data. We did not do any subsequent manipulation or filtering to the data.]
- Thanks for supplying your R code on Github. I saw a summary Rmd file there. Maybe it would be worth considering to include the rendered pdf as a supplement to the manuscript? *[We wrote the RMD is roughly follow the manuscript, so it would be redundant as a supplement in the sense that it reproduces many of the figures.]*
- ll. 197-210: Nice description of the process, but maybe consider to move to supplements

  [This section about DPMMs was removed and the clustering section overall was shortened and adjusted to better justify the approach.]
- l. 235: Very important point, that the slopes within a cluster are identical. Do you consider this when sampling the remaining parameters, that you only sample from the chains with the corresponding fixed slope? Is the RGCA approach in general also applicable for differing slopes?

  [This is not an issue with the newer version of our methodology, since we do not determine the slopes used for sample curves using the clustering. However, in general, RGCA can be used with arbitrary positive slopes.]
- ll. 252-262: consider to move to supplements

  [We felt this description of the TOX21 data provides context and helps motivate the assumption of CA, but we are not opposed to moving it to the SI in a subsequent revision, especially if it is considered common knowledge.]
- l. 289-290: It did not became clear to me, how you arrived at "realistic" Hill parameters, and what "feasible clustering conditionals" means. Please also state how many different mixtures you simulate, with how many chemicals included and how the mixture compositions are defined.

  [The the simulation study has been updated and the text includes more details about the parameter sampling.]

- **Results** l. 317: It would help to reiterate your assumptions here

  [We have expanded our simulation study in a way that is hopefully more relevant and makes the assumptions more clear.]

- Figure 3: Please state n. Would it be feasible to simulate larger mixtures? *[Mixture and sample size has been included in the caption.]*

- l. 336: This is a very interesting finding. It would be nice, if you could plot the fits and the mentioned extrapolations, so one could judge how they differ and which would be more plausible. Include here or in the supplement. *[A comment referencing the plot in the SI has been added.]*

- l. 347: "below" Figure 4? *[This was referencing figure 4. The DPMM has been removed from the manuscript and this section was updated to reflect the new method.]*

- l. 384: Maybe you could draw a dot/ or boxplot showing CRPS score against the number of mixture components and different colors/shapes for the different prediction methods?

  [We feel the updated plots are quite crowded with curves so we added a CAS column to the SI mixture description so that the corresponding scores can be found in the data tables.]

- Figure 6: What are the large circles? replicate 1? It is confusing that they are larger than Replicate 2 and 3 and not mentioned in the legend. In my opinion more fits would deserve to be included in the main part of the manuscript. It would also be interesting to include the CRPS score for the different models in the plot so one could get a feeling for what the numbers mean. Consider to include at least a plot with a very low score/ a medium score and a high score...Include all the remaining fits in the supplement.

  [The plots now show all replicates as equal sized points. We included some additional plots in the main document for comparison.]

- Discussion: l. 380: "Fails with binary and small mixtures": When looking at Supplemental Figure 5, Binary AR2 and AR7, I get the impression, that your RGD approach actually performs not totally bad, since the measured responses fall in the (quite broad) credible interval? What did not become clear to me, why the credible interval is so wide for RGD exclusively, because the clustering cannot be so variable for 2 substances, right? Could you elaborate on this?

  [The variability in these cases is due to the combination of parameter variability and the differences between GCA and IA rather than the cluster variability.]

- l. 422: Workaround would then be back to GCA? *[That is correct. We have modified the algorithm to get rid of this issue to an extent and have updated the methodology section to explore the limits of RGCA in more detail.]*
- ll. 427ff: Can you relate to any exisiting literature on this topic? *[We have added some citations for this problem. In general, such approaches are applied with expert knowledge and we are not aware of a direct comparison to our approach.]*

[Thank you for your review!]
